# Sex/Gender Differences in the Association between Residential Green Space and Self-Rated Health—A Sex/Gender-Focused Systematic Review

**DOI:** 10.3390/ijerph16234818

**Published:** 2019-11-30

**Authors:** Gabriele Bolte, Sarah Nanninga, Lisa Dandolo

**Affiliations:** 1 Department of Social Epidemiology, Institute of Public Health and Nursing Research, University of Bremen, 28359 Bremen, Germany; 2 Health Sciences Bremen, University of Bremen, 28359 Bremen, Germany

**Keywords:** gender, sex, self-rated health, green space, greenness, park, environment, nature, blue space

## Abstract

Though sex/gender is an important social determinant of health, sex/gender inequalities have not been considered comprehensively in environmental health research thus far. The aim of this systematic review was to clarify whether sex/gender theoretical concepts were addressed in studies on the impact of residential green space on self-rated health and whether effect modification by sex/gender was observed. Three electronic databases were searched to identify epidemiological studies on perceived or objective residential green/blue space and self-rated health. Necessary for study inclusion was mentioning at least one keyword for sex/gender in title or abstract, adult study participants and data on self-rated health and on availability and/or use of green/blue spaces. Decisive for study inclusion was consideration of sex/gender differences in the impact of perceived or objective residential green/blue spaces on self-rated health in the analysis and presentation of results. Seven studies were included. They presented an overall positive impact of green space on self-rated health. No consistent sex/gender differences in the impact of green space on self-rated health were found in these studies. However, all studies used a binary operationalization male/female without further theoretical foundation. Research quality could be enhanced by integrating sex/gender-theoretical concepts into study design and interpretation of results.

## 1. Introduction

Sex/gender is a ubiquitous but often ignored social determinant of health [1]. To achieve valid scientific results and to avoid sex/gender bias in research, consideration of both gender relations and sex-linked biology and their entanglement have been recommended [2,3]. The term sex/gender is used throughout this article to emphasize that it is not possible to entirely separate the biological dimensions (“sex”) from the social dimensions (“gender”) in accordance with Springer et al. [4], who conceptualized “sex/gender as a domain of complex phenomena that are simultaneously biological and social” (p. 1818). Though there is a growing awareness of the importance of sex/gender perspectives in several areas of health research, there is still a need to clarify the use of central sex/gender theoretical concepts [3]. In research on environmental health, sex/gender inequalities have thus far not been considered comprehensively [5,6].

In recent years, potential beneficial public health effects of residential green or blue spaces have been an increasing research focus within environmental health research [7,8,9,10,11]. The mechanisms linking green or blue spaces with health are currently a matter of debate [8,12]. Whereas on the one hand health benefits linked with access to green space may be more pronounced among groups with a low socioeconomic position, on the other hand, socially disadvantaged people more often have less access to public green space or the available green space is of poorer quality [10,13]. Exposure differentials in terms of sex/gender disparities in access to environmental goods such as parks have also been shown [14]. Besides socioeconomic position, sex/gender may also modify relationships between green space and health. Both physiological and psychological responses to greenness as well as activity type and frequency of use of green space may differ between females and males [8,10,15]. Therefore, one recommendation of an expert workshop for future epidemiological research in the field of green space and health is to study potential effect modification by sex/gender [8].

For sex/gender-sensitive analyses of health effects of residential environmental goods, the exposure assessment with conceptualization and operationalization of green space or blue space seems to be pivotal. In general, green or blue spaces can be divided into two categories: objectively measured residential greenness or blueness via land use plans or satellite data (e.g., NDVI = Normalized Difference Vegetation Index), or the perceived amount, occurrence or quality of residential green or blue spaces via self-administered questionnaires. Both approaches can lead to different impacts on self-rated health since the objective amount of greenness and blueness in one’s neighborhood can be very different from the personal perceived greenness [16]. It has been suggested that the association between green space and men’s health may be demonstrated accurately by an objectively measured amount of greenness [17]. In contrast, the association between green space and health in women may be better described by subjective indicators such as quality of green spaces, restorative values of natural environments and perceived personal safety [15].

Numerous studies analyzed the physiologically and psychologically restorative effects of nature with self-rated health as outcome [18,19,20,21,22]. Self-rated health has been shown to be a valid measure of physical and mental health in several countries with only slight differences in validity between women and men [23].

To follow the recommendations to further study the complex interrelationships between sex/gender, nature and health [8,17], this systematic review aims to clarify the following:

- whether sex/gender theoretical concepts were addressed in the design of epidemiological studies on the impact of perceived or objective residential green or blue space on self-rated health,
- whether and how sex-linked biology and/or gender were defined and operationalized in these studies,
- whether validity of exposure and outcome metrics were assessed by sex/gender,
- whether sex/gender differences were observed in the association of perceived or objective residential green or blue space and self-rated health,
- whether study results were discussed against the background of sex/gender theoretical concepts.

Unless otherwise stated, the term green space is used in this article as general term for all kinds of greenness or forms of green spaces, objectively or subjectively assessed. The same applies for the term blue space: it is used for all kinds of surfaces of water (e.g., lakes, rivers), objectively or subjectively assessed.

## 2. Materials and Methods

This systematic review follows the Preferred Reporting Items for Systematic Reviews and Meta-Analyses (PRISMA) guidelines for systematic reviews [24] (Supplementary File S1).

### 2.1. Literature Search

The three electronic databases PubMed, Web of Science Core Collection and PsycINFO (via Ovid) were used for the systematic literature search. The applied search syntax consisted of keywords related to sex/gender, self-rated health and green or blue spaces and was conducted on 30 October 2018. It was restricted to articles in the English or German language published between 2000 and 2018. An example of the search syntax applied to title and abstract is shown in Supplementary File S2. Search results were exported to and assessed with EndNote X7.8 (Thomson Reuters, Toronto, ON, Canada) to facilitate the selection process as well as reviewer collaboration.

### 2.2. Selection Process

In a first step, two reviewers independently screened title and abstracts of all results of the literature search against eligibility criteria. In a second step, full texts of all potentially relevant articles were checked for eligibility by two reviewers. Any disagreements about eligibility of a study were resolved by discussion and consultation of a third reviewer if necessary. In a last step, the reference lists of all included articles were screened by one reviewer to identify any further relevant publications not retrieved by the electronic database searches.

### 2.3. Eligibility Criteria

The inclusion or exclusion of studies was carried out in two steps. (1) Necessary for the inclusion of a study was mentioning at least one keyword for sex/gender in title or abstract, presenting adult study participants and providing data on use and/or availability of green or blue spaces and self-rated health. Green or blue spaces could be measured subjectively, objectively or both. Studies fulfilling these criteria were chosen for the full text analysis. (2) Decisive for the inclusion of a study into the review were both the consideration of sex/gender in the impact of perceived or objective residential green or blue space on self-rated health in the analysis and the presentation of sex/gender-related results in either the results section or the discussion. In other words, mere adjustment for sex/gender in multivariate analysis was not sufficient for inclusion in this review. We followed this strategy to make sure that we only included studies that conducted their analysis to find or rule out sex/gender differences in the association of environmental goods and self-rated health.

Exclusion criteria at both stages were samples consisting of only one sex/gender group and study samples consisting exclusively of children. Studies dealing with gardening, stress, anxiety or psychological well-being did not meet the inclusion criteria. Reviews, guides or handbooks were also excluded since only primary articles should be included in systematic reviews.

### 2.4. Evaluation of the Studies

The data extraction was performed by all three authors. Any discrepancy was resolved by discussion between first and last author. Two pre-defined tables were used for data collection: Table 1 contains information about the study design, the study population, the measurement of self-rated health, the measurement of green or blue spaces, the source of the sex/gender data, the operationalization of sex/gender and the terminology used for sex/gender. Table 2 comprises information about the rationale for testing sex/gender differences, the type of analysis testing sex/gender differences, the sex/gender results for both the objectively measured or subjectively perceived green space, and the discussion of the sex/gender results. Supplementary File S3 provides the quotations of the articles used to classify the rationale for testing sex/gender differences and the discussion of sex/gender results.

For the quality assessment of the included studies, a tool for observational studies was applied, which was developed based on the quality checklist proposed by the Effective Public Health Practice Project (EPHPP) [25] and the Strengthening the Reporting of Observational Studies in Epidemiology (STROBE) guidelines for reporting observational studies [26] (Supplementary File S4). The tool consists of a five-item checklist to assess several risks of bias and methodological quality criteria of each study. The quality of the included studies was rated independently by two authors (S.N., G.B.). Disagreements were solved by discussions.

### 2.5. Synthesis of the Results

The results of the included studies were summarized in text form and in Table 2.

## 3. Results

**Figure 1:**
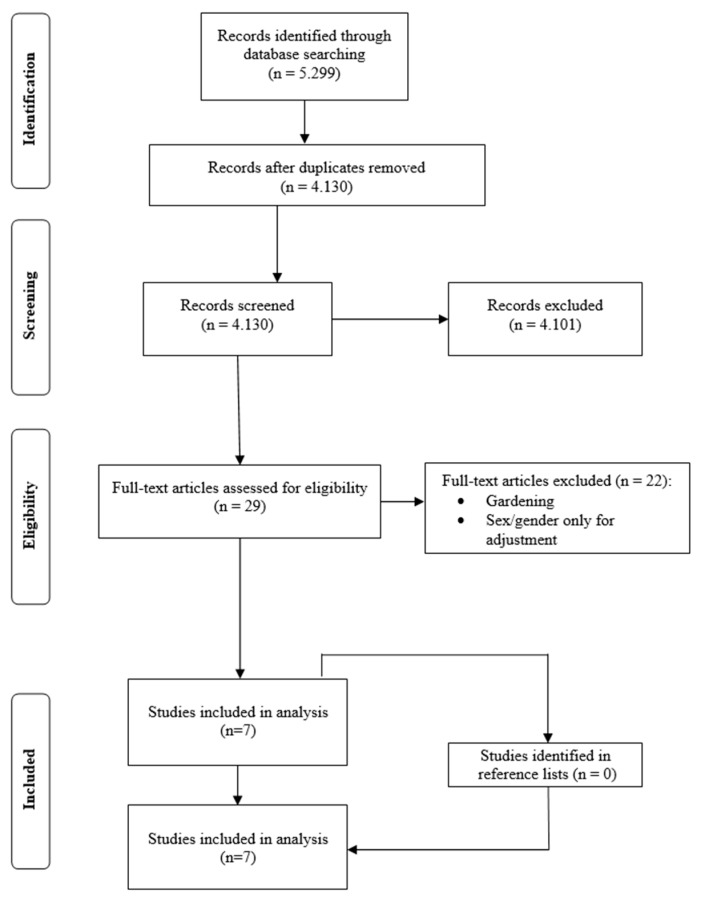
Preferred Reporting Items for Systematic Reviews and Meta-Analyses (PRISMA)-flow diagram of the selection process.

5299 records were identified by the systematic search (PubMed: 2321, Web of Science Core Collection: 2204, PsycINFO: 774). After removing duplicates, 4130 potential eligible articles were screened. The check of titles and abstracts for the necessary inclusion criteria gave 29 articles whose full-texts were assessed for eligibility according to the inclusion criteria. Finally, seven of the 29 studies were included in the analysis of this systematic review and no further studies were found in the reference lists [27,28,29,30,31,32,33]. Two studies presented results from the PHENOTYPE (Positive Health Effects of the Natural Outdoor Environment in Typical Populations in Different Regions in Europe) project [30,31]. As they analyzed different survey periods with different measurements for green spaces both of the articles were included in this systematic review and did not have to be considered as one study. A PRISMA flow diagram of the study selection process is given in Figure 1.

### 3.1. Quality of the Included Studies

Detailed results of the quality assessment are given in Supplementary File S5. Overall, the included studies gained fair results for quality. Selection bias was a problem as almost all of the included studies had a response rate lower than 60%. However, representativeness of the study population and validity or reliability of data collection tools were shown to be valid in all of the included studies. Moreover, all seven studies considered confounders, but reasons were not given. Another aspect of good quality [34] that was not assessed in the quality tool itself was the large sample size in all of the included studies.

### 3.2. General Study Characteristics

All of the seven included studies were cross-sectional with study sizes ranging from 997 [32] to 24,819 [27] participants (see Table 1 for all study sizes). Two studies were conducted in Spain [28,33] and one each in Austria (Graz) [32], Lithuania (Kaunas City) [30], Germany [29] and Sweden [27]. One study combined results from the four cities Barcelona (Spain), Kaunas (Lithuania), Doetinchen (Netherlands) and Stoke-on-Trent (UK) [31]. Studies were published between 2008 and 2017. One study reported results for rural or suburban areas [27], while the remaining six studies tested urban areas.

All of the included studies asked for general self-rated health via a seven- [27] or five- [28,29,30,31,32,33] point-Likert scale in a questionnaire.

In three studies [28,30,31], green space was measured both objectively and subjectively, in one of the studies [32] only subjectively and in the remaining three studies [27,29,33] only objectively. Out of the six studies that measured green space objectively, three of the studies [27,30,31] measured surrounding green space as the percentage of green space in a specific buffer (100 m to 1 km) around the home address using land cover maps. The other three studies [28,29,33] used the Normalized Difference Vegetation Index (NDVI) for evaluating surrounding greenness in buffers of 100 m to 1000 m around the participant’s home address. The subjective measures included self-reports about the perceived amount and quality of green spaces [31,32], self-reports about parks within 10 min walks [28], or self-reports about park usage [30]. Blue spaces were mentioned in two studies [31,33].

The impact of green space on self-rated health was measured by logistic regression models [27,28,29,30,32,33] or multilevel [31] models. Sex/gender differences in terms of effect modification by sex/gender of the association between green space and self-rated health were assessed either by testing for interactions [27,31] or by stratification for sex/gender groups [28,29,30,32], with one study [33] reporting results of both these techniques.

The PHENOTYPE project assessed blue spaces with audit data and with questions about perceived amount and quality of blue spaces in the neighborhood in four cities [31]. The article [31] gave no separate results on the association between blue space and self-rated health. The study in Catalonia measured access to blue space using land cover maps [33]. In the results section, it is stated that residential proximity to blue spaces was not associated with health in this study [33]. Therefore, no further data on sex/gender, blue space and self-rated health are shown in this review.

For further details of the included studies, see Table 1 and Table 2.

### 3.3. Operationalisation of Sex/Gender, Source of Sex/Gender Data, Sex/Gender Terminology and Validity of Exposure and Outcome Metrics in Sex/Gender Groups

Importantly, all of the seven included studies used a binary operationalization of sex/gender, dividing the population into the two groups female and male. Thus, none of the studies acknowledged other sex/gender identities or included other sex/gender dimensions in the analysis.

Additionally, only one study stated that the source of the binary classification male/female was a question asking the participants about their “gender” in a computer-assisted telephone interview [32], none of the other six studies clearly defined the source of the binary sex/gender data. Three studies stated that they used registry information to sample the study population to include equal numbers of women and men [27,30], or numbers according to the age and “sex” structure of the respective district [28]. However, none of these three studies clearly defined if this registry information was also used as the final source for the binary sex/gender variable or whether subjects were additionally asked about their sex/gender group in the respective interviews or questionnaires. Some studies stated that the data in general was collected via personal face-to-face interviews [28,29,31] but did not clarify whether participants were asked about their sex/gender group during the interview or whether the interviewer might have assigned a sex/gender group based on physical appearance. In another study it was not clear whether interviews were conducted face-to-face or via telephone [33]. It is also not clearly described in any of the seven studies whether participants were forced to choose one of the two categories female or male in the questionnaires or interviews or whether they could choose other options.

With respect to terminology, four of the studies used the term “gender” [27,30,32,33] and one of the studies used the term “sex” [28] to refer to the binary classification female/male, while the remaining two studies used the terms “gender” and “sex” interchangeably [29,31]. None of the studies explained why they chose to use either of the terms or both of them, nor did they give definitions of the terms or reflected the usage of them.

According to the guidelines and recommendations of the German Society for Epidemiology [35], all epidemiological studies should present the validity of exposure and outcome metrics according to “sex” and age. However, none of the seven studies discussed whether the exposure measurements for perceived or objective residential green space were equally valid in both included sex/gender groups, nor did they discuss whether the outcome measurements for self-rated health were equally valid.

For a summary of the above inquiries see Table 1.

### 3.4. Sex/Gender Differences in the Association between Objectively Measured Green Space and Self-Rated Health

Out of the six studies that tested the effect of objectively measured green space on self-rated health, three studies found no effect modification by sex/gender: Björk et al. [27] assessed the presence of recreational values within 100–300 m around the residential address via land and vegetation cover data. Overall, the weak evidence for an association between good self-rated health and the number of recreational values within 300 m distance disappeared completely after adjustment for potential confounders. There were no signs of effect modification by sex/gender. Orban et al. [29] found an inverse association of poor self-rated health and higher amount of residential surrounding greenness for 100 m and 1000 m buffers. However, there were no differences in the association between self-rated health and greenness by sex/gender. Ruijsbroek et al. [31] used the Urban Atlas for measuring the amount of green space in three cities in three different countries and the Dutch database Top10NL for the city in the Netherlands. Although the data of the sex/gender analysis was not shown in the article, the authors stated that the association between neighborhood green space and self-rated general health did not differ significantly between men and women in the four cities.

The remaining studies found sex/gender differences in the association between objectively measured green space and self-rated health. Triguero-Mas et al. [33] considered both surrounding greenness via NDVI and access to green space via land cover map data on present green space in a 300 m buffer around the home address. Surrounding greenness was associated with a lower chance for poor health. Access to green space also showed a reduced odds ratio, but not statistically significant. The stratification by males/females revealed an association between surrounding greenness and self-rated health in women, but not in men. However, this difference between the two sex/gender groups should be interpreted with caution, because the odds ratios for less than good self-perceived general health were in the same order of magnitude and there was a wide overlap of the confidence intervals (women: OR 0.89 (95% CI 0.80–0.99), men: OR 0.91 (95% CI 0.81–1.03)). Access to green space was not associated with self-rated health in both males and females.

Dadvand et al. [28] considered both residential surrounding greenness via NDVI and objective residential proximity to green space via utilizing the Parks and Garden Map of Barcelona. They found an overall association of residential surrounding greenness with self-rated health for all investigated buffer sizes (100 m, 250 m, 500 m), whereas objective residential proximity to green space had no significant impact on self-rated health. For residential surrounding greenness, the stratification by male/female yielded the result that only men benefit from surrounding greenness with better self-reported health (men: OR 1.33 (95% CI 1.13–1.57), women: OR 1.07 (95% CI 0.92–1.23)).

Reklaitiene et al. [30] used spatial land covering datasets for Kaunas city to assess green space exposure defined as structured city parks near the participant’s home address. They analyzed three distance categories (<300 m, 300–999 m, ≥1000 m) in combination with categories of self-reported time spent in a park (<4 h/week park use, ≥4 h/week). There was no association between distance to a park and self-rated health in study participants with no park use or use less than 4 h/week. In contrast, among those study participants with a park use of ≥4 h/week, increasing distance to a park was associated with an increase in the prevalence of poor self-rated health. Stratification by men/women showed this relationship only in women (park use ≥4 h/week, distance to the park 300–999 m: OR for poor self-rated health 1.89 (95% CI 1.17–3.07), ≥1000 m distance: OR 1.68 (95% CI 0.81–3.48), *p* for trend = 0.041), but not in men (park use ≥4 h/week, distance to the park 300–999 m: OR for poor self-rated health 0.88 (95% CI 0.45–1.74), ≥1000 m distance: OR 1.72 (95% CI 0.69–4.29), *p* for trend = 0.42). However, in the exposure category distance ≥1000 m, the odds ratios for men and women were in the same order of magnitude and there was a wide overlap of the confidence intervals.

For a summary of the above results see Table 2.

### 3.5. Sex/Gender Differences in the Association between Subjectively Assessed Green Space and Self-Rated Health

Three studies reported subjectively assessed data on green space [28,31,32]. Two of these studies found no effect modification by sex/gender for the association of subjectively perceived green space and self-rated health: Ruijsbroek et al. [31] asked their participants about the amount of perceived green space in the neighborhood using a 5-point Likert scale. The authors stated that the association between perceived neighborhood green space and self-rated health did not differ significantly between men and women. Data were not shown in the article.

In the study by Stronegger et al. [32], the perception of green space in the neighborhood was part of an indicator for environmental characteristics of the living quarter. Analysis results were given for this indicator “social-environmental quality”. In both, men and women, high perceived social-environmental quality was associated with good self-rated health (men: OR 1.99 95% CI 1.18–3.34, women: OR 1.81 (95% CI 1.05–3.10)); thus, there was no effect modification by sex/gender.

The study by Dadvand et al. [28] gained data on the subjective residential proximity to green space by asking the participants whether or not they have a park within ten minutes’ walk from their home. Overall, subjective proximity to green space was positively associated with self-rated health. Stratification by male/female yielded a statistically significant association with good self-rated health in women (OR 1.41 (95% CI 1.07–1.86)), while a similar association for men did not reach statistical significance (OR 1.32 (95% CI 0.98–1.78)). Thus, this indication of an effect modification should be interpreted with caution.

For a summary of the above results see Table 2. The results of Reklaitiene et al. [30], who analyzed a combination of objective and subjective data on green space, are described in paragraph 3.4.

### 3.6. Consideration of Other Sociodemographic or Socioeconomic Dimensions

All studies adjusted for other sociodemographic or socioeconomic dimensions as potential confounders, such as age, ethnicity, education, marital status, household composition, home­ownership, employment status, problems with paying bills, type of health insurance, neighborhood socioeconomic status.

None of the studies analyzed the impact of combinations of sex/gender and further sociodemographic or socioeconomic dimensions on the association of green space and self-rated health.

### 3.7. Rationale for Testing Sex/Gender Differences and the Usage of Sex/Gender-Theoretical Concepts in the Discussion of the Results

Out of the seven studies, three studies [29,30,32] did not give any rationale for testing sex/gender differences. The same three studies did not discuss the sex/gender results regarding the association of green space and self-rated health.

Out of the remaining four studies, three studies [27,31,33] stated that their rationale for testing sex/gender differences was previous research showing an effect modification by sex/gender in the respective green space-health associations, thereby all three studies referred to similar previous research [15,18,19,20]. The remaining study [28] claimed that the rationale for testing sex/gender differences is an assumption of a possible effect modification by sex/gender although an evidence for such a modification would be non-existent.

In the discussion of the results on potential effect modification by sex/gender, two studies [31,33] presented care activities as a possible reason for sex/gender differences, assuming that women spend more time in parks than men because they spend more time looking after children and older people. Another reason, stated in two studies [27,31], is that the radius of action in residential environments is still smaller for women than for men, as women apparently fulfill social roles that are more locally oriented, claiming that women’s health is therefore more strongly influenced by the characteristics of their neighborhoods than men. One study [28] suggests that the usage of green spaces differs between men and women, claiming that men more frequently use green spaces and are more physically active in green spaces. Despite these specific arguments regarding the sex/gender differences, none of the studies discusses any underlying sex/gender theoretical concepts in more detail.

For a summary of the above observations see Table 2 and for a closer inspection of all relevant text passages regarding either the rationale for testing sex/gender differences or the discussion of sex/gender results in the respective studies see Supplementary File S3 with the quotations.

## 4. Discussion

In this systematic review, we analyzed epidemiological studies that explored effect modification by sex/gender of the association between perceived or objective residential green space and self-rated health. The purpose of this review was to comprehensively examine whether and how the identified seven studies applied sex/gender theoretical concepts in study aim, study design (definition and operationalization of sex/gender, use of validated survey instruments, statistical analyses) and discussion of results.

### 4.1. Consideration of Sex/Gender-Theoretical Concepts in Study Design

None of the studies included in this review referred to any of the central sex/gender concepts [2,3,36] in their respective introductions. The only rationale given for testing effect modification by sex/gender were references to previous studies that showed differences between women and men in the association between green space and health. As a consequence, all seven studies used only a binary operationalization of sex/gender with the categories female and male, without acknowledging other possible sex/gender categories or allowing for a more complex description of sex/gender through multiple relevant dimensions within a comprehensive sex/gender concept. It has been repeatedly discussed that the binary operationalization male/female assumes homogeneous groups and does not adequately consider multidimensionality, context dependence and dynamics according to time and place of sex/gender [37,38]. Another point of criticism is that most studies gave no definite description on how the binary sex/gender data was obtained, thus, it is not clear whether registry information, self-report or assignments through interviewers led to the female/male classifications. Additionally, it is unclear whether participants were forced to choose one of the two categories, even if they would not identify themselves either male or female.

Those studies using already existing data of a health survey did not have the possibility to take complexity of sex/gender at the step of data collection into account, as has been recommended for health surveys [39,40,41]. Nevertheless, the concept of intersectionality could have been integrated in data analyses by exploring interactions between sex/gender and further social dimensions to assess the simultaneous influence on health [42,43,44]. However, in all studies, sociodemographic or socioeconomic variables were conceptualized as potential confounders and used for adjustment in statistical analyses on sex/gender, green space and self-rated health.

Furthermore, the authors of the included studies did not comment on validity of the applied exposure and outcome measurements regarding females and males as has been recommended [35].

Finally, none of the studies clarified their use of the terms sex and/or gender, and an interchangeable use of these terms was observed in two studies [29,31], leading to a conceptual muddle of the terms “gender” and “sex” as criticized before [2,45]. In recent years, an increasing number of journals has included sex/gender-specific guidelines in their instructions for authors to encourage sex/gender-sensitive data analysis and reporting [46,47]. These guidelines refer to, e.g., correct use of the terms “sex” and “gender”, reporting of “sex and/or gender” of study participants, description of methods used to determine sex/gender and consideration of heterogeneity of associations in data analyses.

Out of the six different journals in which the seven studies included in this review were published, only one, the Scandinavian Journal of Public Health, in which the study by Reklaitiene et al. [30] was published, advises authors to follow the “Recommendations for the Conduct, Reporting, Editing, and Publication of Scholarly Work in Medical Journals” by the International Committee of Medical Journal Editors [48]. In these recommendations, authors are encouraged to reflect on their usage of the terminology of “sex” (defined as biological factors) and “gender” (defined as identity, psychosocial or cultural factors), to describe the methods used to determine “sex” and “gender”, to stratify the analysis by “sex” and to discuss the influence of “sex and/or gender” on the findings. It is, however, not clear whether Reklaitiene et al. [30] were already advised to follow these recommendations at the time of manuscript preparation and publication in the year 2014.

### 4.2. Sex/Gender Differences in the Association of Green Space and Self-Rated Health

Overall, the included seven studies showed a positive impact of green space on self-rated health, which is in line with previous reviews [7,10,17]. However, using a binary operationalization, no consistent sex/gender differences in the impact of green space on self-rated health were found. For both strategies to define exposure, objective and subjectively perceived green space, four studies [27,29,31,32] reported no effect modification by sex/gender, while the remaining studies reported either positive outcomes only for men ([28] in case of objective green space measure) or only for women [30,33] and [28] in case of subjective green space measure. Additionally, a closer look at some results [28,33] showed that the presented differences between effect estimates for men and women were very small and confidence intervals overlapped widely. As only three of the included studies presented results for both perceived and objective green space within the same study, an adequate comparison of these two strategies of exposure assessment with regard to relevance for effect modification by sex/gender was not possible. Hence, overall, the evidence on potential effect modification by sex/gender on the association of green space and self-rated health was inconclusive. Moreover, no studies with analysis results on sex/gender differences in the association of blue space and self-rated health could be identified.

According to Stafford et al. [49], sex/gender differences in associations between characteristics of the residential environment, work conditions and health may be cohort- or context-specific; thus, comparisons between different study population of various countries should be interpreted with caution. Therefore, the explanatory approaches discussed in the studies are of particular interest.

### 4.3. Consideration of Sex/Gender-Theoretical Concepts in Discussion of Results

Previous research referred to several explanatory approaches for potential sex/gender differences in the association of residential environment and health: exposure differentials, vulnerability differentials (physiological or psychological pathways), different amount of time spent at home and in the immediate surrounding due to gender roles (care responsibilities) and differences in activity type and frequency of green space use due to, e.g., perceived safety or quality of green space [8,10,14,15,17,49,50]. Besides these aspects, a methodical issue might be sex/gender differences in self-report of residential green space or health [15].

The arguments of the studies included in this review are in line with the previous reasoning: especially gender roles and their implications for care responsibilities, time spent at home or in the neighborhood and the radius of action in the residential environment were discussed [27,31,33]. Nevertheless, none of the studies explicitly referred to one of the central sex/gender-theoretical concepts in health research [3]. The reference to gender roles and the treatment of women and men, respectively, as homogeneous groups might be interpreted according to Hammarström et al. [3] as a static difference perspective with a dichotomous variable for sex/gender on an individual level. Annandale and Hunt [51] criticized the analysis of social roles and status as properties of individuals which affect health as a traditional methodological approach and proposed to put more emphasis on social change over time in the gender order at individual and structural level. Hammarström et al. [3] emphasized that the static difference perspective implicates the risks of overemphasizing differences between women and men and of generalization of differences to all groups of women and men independent of context.

### 4.4. Strengths and Limitations

This systematic review and the included studies both have some limitations.

First of all, only a small number of studies, i.e. seven, could be included and all of these studies had a cross-sectional design. Thus, only associations between green space and self-rated health at one point in time could be studied. Besides temporal changes of gender roles and sex/gender impacts, especially the self-rated health status could change over time and be influenced by far-reaching experiences such as a divorce or job loss or a change in lifestyle behavior such as quitting smoking or use of health care [52].

Only two of the included studies [29,31] reported on quality of green space. Quality characteristics of green spaces like walkability, safety, aesthetics or park facilities are important factors for the prediction of use of green space, especially when it comes to sex/gender differences [17,50,53]. More studies providing evidence about the perception of quality of green space would have been preferable.

The question for self-rated health was a single item of a 5- or 7-point-Likert scale in all of the included studies. Self-rated health is widely accepted as valid predictor of morbidity and mortality [54,55] and only slight differences in validity between women and men have been observed [23]. However, different styles in reporting health may occur across countries [55] and between men and women [15]. None of the included studies commented on validity of the applied survey instruments. Furthermore, subjective health assessment does not only reflect a respondent’s biological dimension of health, but also the psychological and social dimensions [54]. It was therefore in some cases hard to distinguish between studies assessing specifically mental health or general self-perceived health during the search process.

All of the included studies had different approaches to assess green spaces. Hence, a meta-analysis was not possible. The three studies [28,29,33] working with the NDVI used different buffers around the residential address ranging from 100 m to 1000 m, the three other studies with objective measures [27,30,31] worked with different land cover maps. The four studies [28,30,31,32] presenting results for subjectively measured green space asked study participants for example whether there was a park 10 min away from home [28], frequency of park use [30] or perceived amount of greenness [31]. Additionally, the surveys took place at different times with even different seasons. For example, one survey took place from May to October [31], another survey from September to October [32]. When measuring the amount of green space or the neighborhood appearance in terms of green and blue, the time of the year is an important factor that might bias the results of self-reports.

Another limitation could be that we might have missed some studies that tested for sex/gender differences, e.g., through post-hoc sex/gender-stratified analysis, yet did not mention these sex/gender analyses in their title or abstract, as we only searched for studies that used at least one keyword for sex/gender in title or abstract.

The major strengths of this review are that the focus was on up-to-date sex/gender-theoretical concepts and that a rigorous methodology for systematic reviews was applied.

## 5. Conclusions

This systematic review showed that only very few studies tested effect modification by sex/gender in the association of green space and self-rated health. The seven included studies gave no consistent pattern of sex/gender differences in the association of subjectively perceived or objectively measured residential green space and self-rated health. However, all studies used only a binary operationalization of sex/gender, assuming static differences between women and men. Neither in the study rationale, study design nor in the discussion of results did the seven studies refer to current sex/gender-theoretical concepts. Obviously, as stated before by Hammarström and Hensing [56], even in this research area of environmental health, there is a potential to improve the use of sex/gender theories and to consider the complexity of sex/gender in epidemiological research. Therefore, future research should refer to sex/gender-theoretical concepts in the study design, collect data on several dimensions of sex/gender to allow for more sophisticated statistical analyses of potential exposure variation and effect modification by sex/gender and avoid inaccurate sex/gender stereotypes in the interpretation of results.

## Figures and Tables

**Table 1:** Characteristics of the included studies.

Study Publication	Study Design	Study Population	Measurement of Self-Rated Health	Measurement of Green Space	Measurement of Blue Space	Operationalisation of Sex/Gender	Source of Sex/Gender Data	Terminology
Björk et al. [27]	Cross-sectional, Public Health Survey in southern Sweden, 2004	*N* = 24,819, female = 54.3%, suburban/rural, Sweden	7-point-likert scale (very poor to very good)	Objective: Land and vegetation cover (CORINE), 100 m–300 m buffer	no	binary	source not clearly defined, but registry information was initially used to contact equal numbers of women and men via a mailed questionnaire	gender
Dadvand et al. [28]	Cross-sectional, Health Survey of Barcelona, 2011	*N* = 3461, female = 52.1% urban, Spain	5-point-likert scale (excellent to bad)	Objective: NDVI, 100 m–500 m buffer; land cover map, 300 m, Subjective: Park within 10 min walk (self-report)	no	binary	source not clearly defined, but registry information was initially used to select subjects for a face-to-face interview in a way to represent age and sex structure of districts	sex
Orban et al. [29]	Cross-sectional, Heinz Nixdorf Recall Study, 2000–2003	*N* = 4480, female = 49.7%, urban, Germany	5-point-likert scale (very good to very poor)	Objective: NDVI, 100 m–1000 m buffer	no	binary	source not clearly defined, only general statement that data was obtained through personal interviews and questionnaires	gender and sex interchangeably
Reklaitiene et al. [30]	Cross-sectional, PHENOTYPE, 2006-2008	*N* = 6944, female = 54.6%, urban, Lithuania	5-point-likert scale (very good to very poor)	Objective: Land cover map, <300 m, 300 m–999 m, ≥1 km Subjective: Park use (self-report)	no	binary	source not clearly defined, but registry information was initially used to draw a random sample stratified by gender and age, data was obtained through self-reported questionnaires	gender
Ruijsbroek et al. [31]	Cross-sectional, PHENOTYPE, 2013	*N* = 3771, female = 55.5%, urban, Spain, Lithuania, Netherlands, United Kingdom	5-point-likert scale (excellent to poor)	Objective: Land cover map (Urban Atlas) Subjective: Perceived amount and quality of green space (self-report)	yes	binary	source not clearly defined, only general statement that data was obtained through face-to-face interviews or a postal questionnaire	gender and sex interchangeably
Stronegger et al. [32]	Cross-sectional, 2005	*N* = 997, female = 50.8%, urban, Austria	5-point-likert scale (very good to very bad)	Subjective: Perceived amount of green space as part of environmental quality	no	binary	question about gender was asked in a computer-assisted telephone interview	gender
Triguero-Mas et al. [33]	Cross-sectional, Catalonia Health Survey ESCA, 2010-2012	*N* = 8793, female = 50.1%, urban, Spain	5-point-likert scale (excellent to bad)	Objective: NDVI, 300 m buffer; land cover map, 300 m	yes	binary	source not clearly defined, only general statement that data was obtained through interviews	gender

Abbreviations: CORINE = Coordination of Information on the Environment; ESCA = Enquesta de Salut de Catalunya; NDVI = Normalized Difference Vegetation Index; PHENOTYPE = Positive Health Effects of the Natural Outdoor Environment in Typical Populations in Different Regions in Europe.

**Table 2:** Consideration of sex/gender theoretical concepts and results of sex/gender analysis.

Study Publication	Rationale for Testing Sex/Gender Differences	Analysis of Sex/Gender Differences	Results for Objectively Measured Green Space	Results for Subjectively Perceived Green Space	Discussion of Sex/Gender Results
Björk et al. [27]	Previous research on effect modification by sex/gender	Regression analysis, Test for interaction by sex/gender	No effect modification	Not applicable	Sex/gender and radius of action in residential environments
Dadvand et al. [28]	Assumption of effect modification by sex/gender	Regression analysis, Stratification by sex/gender	Residential surrounding greenness within 250 m buffer: Positive association with good self-rated health in men, but not in women	Subjective proximity to green spaces: Positive association with good self-rated health in women, In men OR in the same order of magnitude, but not statistically significant	Sex/gender and green space usage
Orban et al. [29]	Not specified	Regression analysis, Stratification by sex/gender	No effect modification	Not applicable	Not specified
Reklaitiene et al. [30]	Not specified	Regression analysis, Stratification by sex/gender	Park use < 4 h/week: No effect modification Park use ≥ 4 h/week: association of distance to park with poor self-rated health in women, In men OR in the same order of magnitude in highest distance category, but not statistically significant		Not specified
Ruijsbroek et al. [31]	Previous research on effect modification by sex/gender	Multilevel regression analysis, Test for interaction by sex/gender	No effect modification	No effect modification	Sex/gender roles (care activities, radius of action in residential environments)
Stronegger et al. [32]	Not specified	Regression analysis, Stratification by sex/gender	Not applicable	No effect modification	Not specified
Triguero-Mas et al. [33]	Previous research on effect modification by sex/gender	Regression analysis, Test for interaction by sex/gender and stratification by sex/gender	Surrounding greenness within 300 m: negative association with poor self-rated health in women, in men OR in the same order of magnitude, but not statistically significant; Access to green space: no effect modification	Not applicable	Sex/gender roles (care activities)

## Supplementary Materials

The following are available online at https://www.mdpi.com/1660-4601/16/23/4818/s1, Supplementary File S1: PRISMA-Statement: Preferred Reporting Items for Systematic Reviews and Meta-Analyses, Supplementary File S2: Sample search string for PubMed MEDLINE, Supplementary File S3: Text citations of rationale for testing sex/gender differences and discussion of sex/gender results, Supplementary File S4: Quality assessment tool for quantitative, observational studies, Supplementary File S5: Results of the quality assessment.
